# Automated fitting of transition state force fields for biomolecular simulations

**DOI:** 10.1371/journal.pone.0264960

**Published:** 2022-03-10

**Authors:** Taylor R. Quinn, Himani N. Patel, Kevin H. Koh, Brandon E. Haines, Per-Ola Norrby, Paul Helquist, Olaf Wiest

**Affiliations:** 1 Department of Chemistry and Biochemistry, University of Notre Dame, Notre Dame, Indiana, United States of America; 2 Early TDE Discovery, Early Oncology, Oncology R&D, AstraZeneca, Boston, Massachusetts, United States of America; 3 Department of Chemistry, Westmont College, Santa Barbara, California, United States of America; 4 Data Science and Modelling, Pharmaceutical Sciences, R&D, AstraZeneca Gothenburg, Mölndal, Sweden; 5 Lab of Computational Chemistry and Drug Design, School of Chemical Biology and Biotechnology, Peking University, Shenzhen Graduate School, Shenzhen, China; Faculdade de Ciências da Universidade de Lisboa, PORTUGAL

## Abstract

The generation of surrogate potential energy functions (PEF) that are orders of magnitude faster to compute but as accurate as the underlying training data from high-level electronic structure methods is one of the most promising applications of fitting procedures in chemistry. In previous work, we have shown that transition state force fields (TSFFs), fitted to the functional form of MM3* force fields using the quantum guided molecular mechanics (Q2MM) method, provide an accurate description of transition states that can be used for stereoselectivity predictions of small molecule reactions. Here, we demonstrate the applicability of the method for fit TSFFs to the well-established Amber force field, which could be used for molecular dynamics studies of enzyme reaction. As a case study, the fitting of a TSFF to the second hydride transfer in *Pseudomonas mevalonii* 3-hydroxy-3-methylglutaryl coenzyme A reductase (*Pm*HMGR) is used. The differences and similarities to fitting of small molecule TSFFs are discussed.

## Introduction

Understanding how enzymes achieve their catalytic function is one of the grand challenges of chemistry and biology. Studying enzymes using computational methods has produced highly impactful work, as highlighted by the award of the Nobel Prize in 2014 [1] for the development of multiscale methods such as the Quantum Mechanics/Molecular Mechanics (QM/MM) method [2]. Because enzymes consist of tens of thousands of atoms, using even low level electronic structure methods is cost prohibitive for the full system. Furthermore, extensive sampling of the conformational space, e.g. by molecular dynamics simulation at the necessary level to obtain physically meaningful results. To enable such simulations, a range of classical force fields that approximate atoms and bonds as masses connected by springs have been developed [3, 4]. The accuracy of these simulations is dependent on the quality of the force field used [5]. As a result, extensive validation studies of the force field functional form as well as the parameters themselves have been performed [6, 7].

The use of machine learning (ML) methods in science and technology has expanded exponentially in recent years, in part due to the rapid expansion in computational power and available datasets. In chemistry, applications of ML range from basic research through material research [8, 9] to drug discovery [10] and force field and PEF parameterization [8, 11–15]. Even though the development of ML methods for the treatment of enzymatic reactions provides an alternative to the computationally expensive QM/MM methods, there have been comparatively few ML applications for large biomolecular systems. One reason is that because large training sets are needed to fit the ML PEF, only small molecules or very small subsections of an enzyme, but not the entire system, can be fitted. One possible solution is to combine ML PEFs with classical force fields in a hybrid approach, where two very different functional forms (at least one of which is not likely to be physically meaningful) are mixed. This is because the functional form of a ML PEF is not constrained to a pre-defined functional form, e.g. the one of a classical force field that has been extensively validated. The effects of this mixing of different energy functions has not yet been systematically explored for the case of ML PEF, but has been the topic of considerable work for the case of hybrid QM/MM methods where the problem of boundary atoms described by the classical and quantum Hamiltonians are well known [16–18].

**Fig 1:**
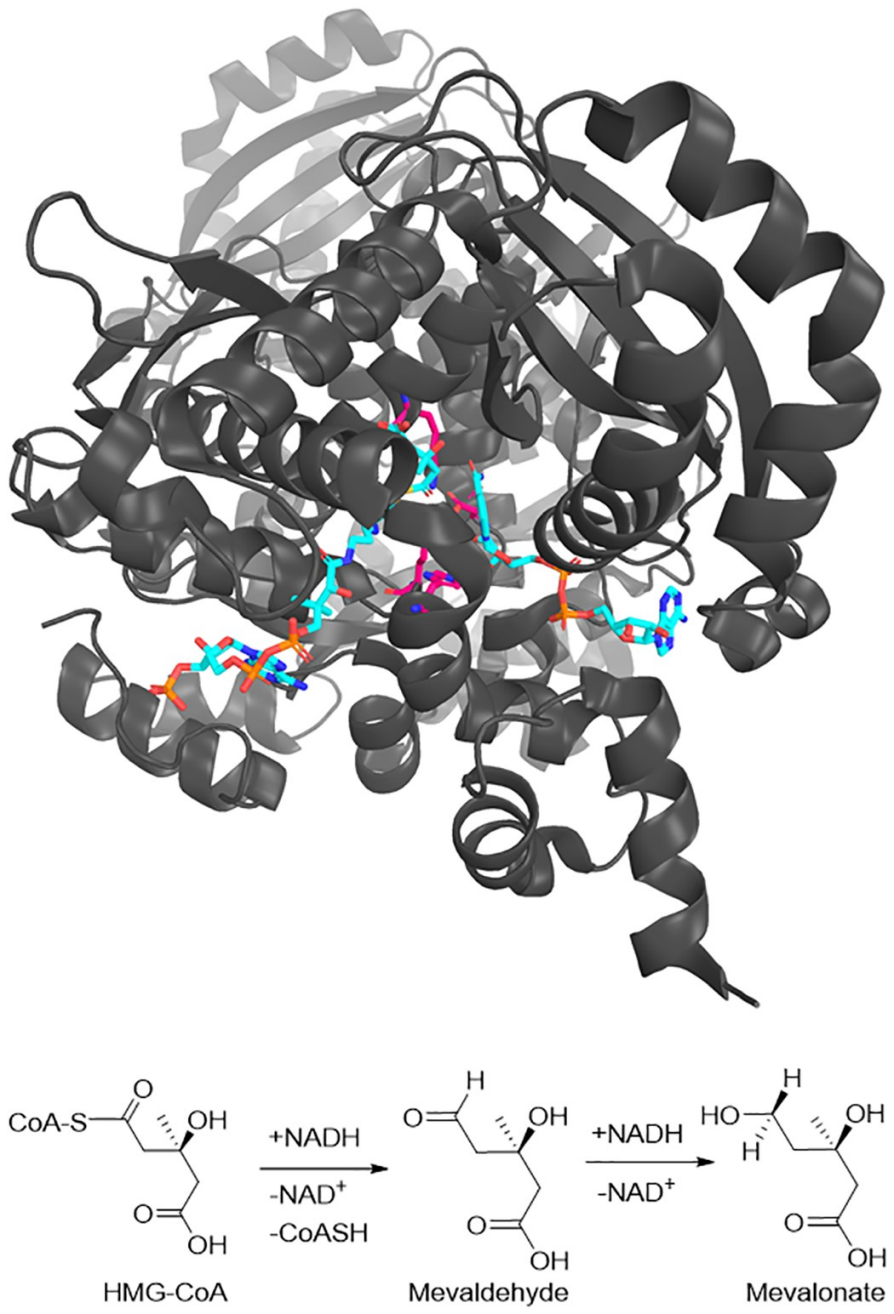
Structure of *Pm*HMGR (pdb code 1QAX, 2.80 Å resolution). The crystallized substrate (HMG-CoA) and cofactor (NAD), shown in cyan, bound to the active site of HMG-CoA reductase (top). Double hydride transfer catalyzed by *PmHMGR* (bottom).

Here, we propose an alternative approach where a consistent energy function is used for the entire system for the study of reactions in biomolecular systems, as exemplified by the structure of *Pseudomonas mevalonii* 3-hydroxy-3-methyl glutaryl-CoA Reductase (*Pm*HMGR) shown on top of Fig 1, which catalyzes the double hydride transfer to convert HMGCoA to mevalonate as shown on the bottom of Fig 1. In this approach, the vast majority of the system (shown in grey in Fig 1) is described by extensively validated classical force fields. These cannot describe the substrate, cofactor, or residues at the transition state of the reaction (shown in color in Fig 1). Thus, the subset of the structure that includes the bond breaking and making atoms as well as key active site residues and cofactors are described by a transition state force fields (TSFF) [19, 20] that has the functional form of an extensively validated classical force field (in the present case, Amber) [21]. Though not formally a ML approach, the philosphy is reminiscent to transfer learning in that the functional form and extensively validated parameters of a classical force field are used and retrained for a subset of the structure that cannot be described by the original PEF. The parameters needed to describe the transition state can then be fitted using a smaller training set by the quantum-guided molecular mechanics (Q2MM) method that was originally developed for the parametrization of small molecule force fields, especially for the prediction of stereoselectivity in transition metal catalyzed reactions [22–24]. In these cases, TSFFs have been demonstrated to be highly accurate with unsigned errors of 2–3 kJ/mol over hundreds of examples from many different reaction types [25, 26].

TSFFs are conceptually related to the empirical valence bond [27, 28] and related methods [29, 30] in that they use classical force fields to describe transition states, but they have some key differences because (i) only a single PEF rather than a mix of reactant and product PEFs is used, (ii) the PEF is parameterized at the transition state rather than describing the transition state as a mix of ground state PEFs and, (iii) no empirical data is used in the fit, making the method truly predictive.

When fitting predefined functional forms to reproduce training data from appropriate electronic structure methods, both linear and non-linear regression algorithms have been used. A genetic algorithm was used to optimize a polarizable force field from ab initio QM data [31] as well as the parameterization of reactive force fields [32]. The Parsely force field for small molecules uses QM data for parameterization of an Amber-lineage with SMIRNOFF atom specification [33]. Similarly, the Amber-15 Force Balance force field [34] for use with the TIP3P-Force Balance water model [35] is fitted using a weighted least-squares method. The AMOEBA-2013 force field also was optimized using automated techniques to obtain a general polarizable protein force field [36]. However, all of these studies concern ground state force fields that are not able to describe bond breaking and making steps of an enzymatic reaction where a TSFF is needed.

Q2MM is one of the best established [22] automated fitting procedures for the parameterization of both ground state and transition state force fields (TSFF). It has been used extensively for the development of TSFF for the prediction of stereoselectivity of small molecule reactions [23, 24, 37, 38]. To the best of our knowledge, the only application of Q2MM to biomolecular systems is a simple version of a TSFF for transition state docking of small molecular drugs to P450 enzymes to identify potential sites of hydroxylation [39, 40], but no further studies exploring the enzyme flexibility using the TSFF were performed. In addition, the code used for this fitting procedure is not widely available, is not compatible with more recent versions of the Amber code and does not contain more recent innovations in the Q2MM method [24, 41].

Q2MM uses training data from electronic structure (usually density functional theory) reference calculations to parameterize molecular mechanics TSFF based on the PEF with the functional form of a classical force fields. The details of this process for asymmetric catalysis by small molecules, where MM3* is used, have been covered elsewhere [25, 26, 42] and will not be elaborated on here. We will focus the application of the Q2MM method to derive TSFFs of a predefined functional form compatible with the Amber-family force fields for biomolecular systems and will touch on the differences to the fitting of small molecule systems. We will also discuss the interfacing of the Q2MM tools to the Amber suite of molecular dynamics programs and demonstrate this workflow for the case of a TSFF for the second hydride transfer of *Pm*HMGR, shown in Fig 1 bottom.

## Fitting methods

Q2MM fits the FF parameters by minimizing the value of objective or penalty function,

$$\chi^{2}=\sum_{i}{w_{i}}^{2}{\left(x_{i}^{0}-x_{i}\right)}^{2}$$

where $x_{i}^{0}$ is the reference data point, *x*_*i*_ is the FF data point, and *w*_*i*_ is the weight for that data point. The minimization step in the parameter space is calculated using gradient-based method such as the Newton-Raphson technique, as well as the simplex method [43]. The gradient-based method is general and utilizes the Jacobian matrix *J* where

$$J_{ij}=\frac{\partial x_{i}}{\partial p_{j}}$$

and *p*_*j*_ is j-th parameter, which is calculated in many programs using numerical differentiation and therefore the rate-determining step. Thus, the simplex method is often used to avoid the high cost of numerical derivatives [44]. The simplex method in Q2MM is modified to move toward the best point(s) in the parameter space using a bias of reflection point [43]. The modified simplex method has shown to have faster convergence than the Raphson-type methods up to ca. 40 parameters [43]. Thus, it is used to optimize a medium-sized parameter set or a subset of the larger parameter set.

Q2MM, unlike most traditional methods for fitting system-specific FF parameters [35, 45, 46], uses the Hessian Matrix for the fitting of force constants of bonded parameters with geometric data for reference structures [43, 47, 48]. The Hessian matrix is the second partial derivative of the energy with respect to the xyz coordinates of atoms, which gives the matrix size of 3N x 3N where N is the number of atoms. It can be obtained by appropriate electronic structure calculations of suitable model systems including, in the case of the large biomolecular systems discussed here, QM/MM calculations. In the latter case, the calculation of the Hessian matrix’s eigenvalues and eigenvectors provides information on the vibrational frequencies and normal modes, respectively. Normally, eigenvalues of the Hessian matrix are positive, but at the transition state geometry, the eigenvalues contain one negative value with its eigenvector representing the reaction vector. By providing Hessian matrix information in the objective function, Q2MM uses information on both the transition state geometry and the shape of the potential energy surface around it when fitting the FF parameters. However, because Q2MM fits these parameters to represent the transition state, which is a saddle point, as a minimum on the potential energy surface, the matrix element that corresponds to the negative eigenvalue is altered during the fitting process [41]. This leads to an increase of the objective function value.

To address this and the fact that the algorithms in most molecular force field-based programs [49] are designed to optimize towards minima rather than transition states, a small modification to Q2MM is made. Traditionally, in the cartesian Hessian fitting method, all indices of the Hessian matrix are accounted for in the objective function with respect to the reference values. However, different weights are assigned to each element of the Hessian matrix to correctly represent the transition state as a minimum. The indices of Hessian matrix are given a weight of 0.0 to 1–1 interactions (Hessian elements where both cartesian coordinates are for the same atom), 0.031 to 1–2 and 1–3 interactions (atoms separated by 1 or 2 bonds, respectively), 0.31 to 1–4 interactions (atoms separated by 3 bonds, *i*.*e*., the terminal atoms of torsions) and 0.031 to all other interactions (atoms separated by >3 bonds) [50]. The cartesian Hessian matrix fitting method is used for large molecule systems such as an enzyme, where only one reference structure is used to fit the parameters.

Alternatively, users can use the eigenmode fitting method in Q2MM. In this method [41], the reference Hessian matrix *H* = *V*^*T*^*EV* is decomposed into eigenvector *V* and eigenvalue *E*. Then the objective function includes the calculated eigenvalue matrix *E*′ where *E*′ = *VH*′*V*^*T*^ and *H*′ is the Hessian matrix of the FF calculated Hessian matrix. By preserving the original eigenvector *V*, all of the originally positive eigenvalues are preserved and only the negative eigenvalue is converted into a positive value by zeroing the weight of the eigenvalue to represent a transition state as a minimum. It should be noted that this inversion of the potential energy surface in the reaction coordinate is done to allow the use of simple energy minimization techniques available in all force field packages to locate the stationary point. However, it is not absolutely required and alternative approaches have been developed [51].

## The Q2MM flow scheme

The following parameterization scheme is specific towards the implementation of the Amber20 [52] interface of Q2MM and its use for large biomolecular systems. Details of the method regarding parameterization of TSFF for small molecule asymmetric catalysis using the MM3* force field in Macromodel have been documented elsewhere [26]. Here, we demonstrate that the Q2MM method is applicable for the fitting force fields for enzyme reactions using the Amber family of force fields. As an example of using Q2MM for biomolecular systems, the development of a TSFF for the second hydride transfer transition state of *Pm*HMGR [53–55], shown in Fig 1, will be discussed. Examples of the files discussed in this section as well as the final TSFF are given in the Supporting Information. The Q2MM code itself, which contains the interface to the Amber Suite of programs, and several published TSFFs are freely available on the Q2MM/CatVS github repository (github.com/q2mm). In this section, file types denotes as.filetype refer to files of the commercial software such as Amber [52] or Gaussian [56] while file name in all-caps (such as FFLD) refer to files used in the Q2MM code.

**Fig 2:**
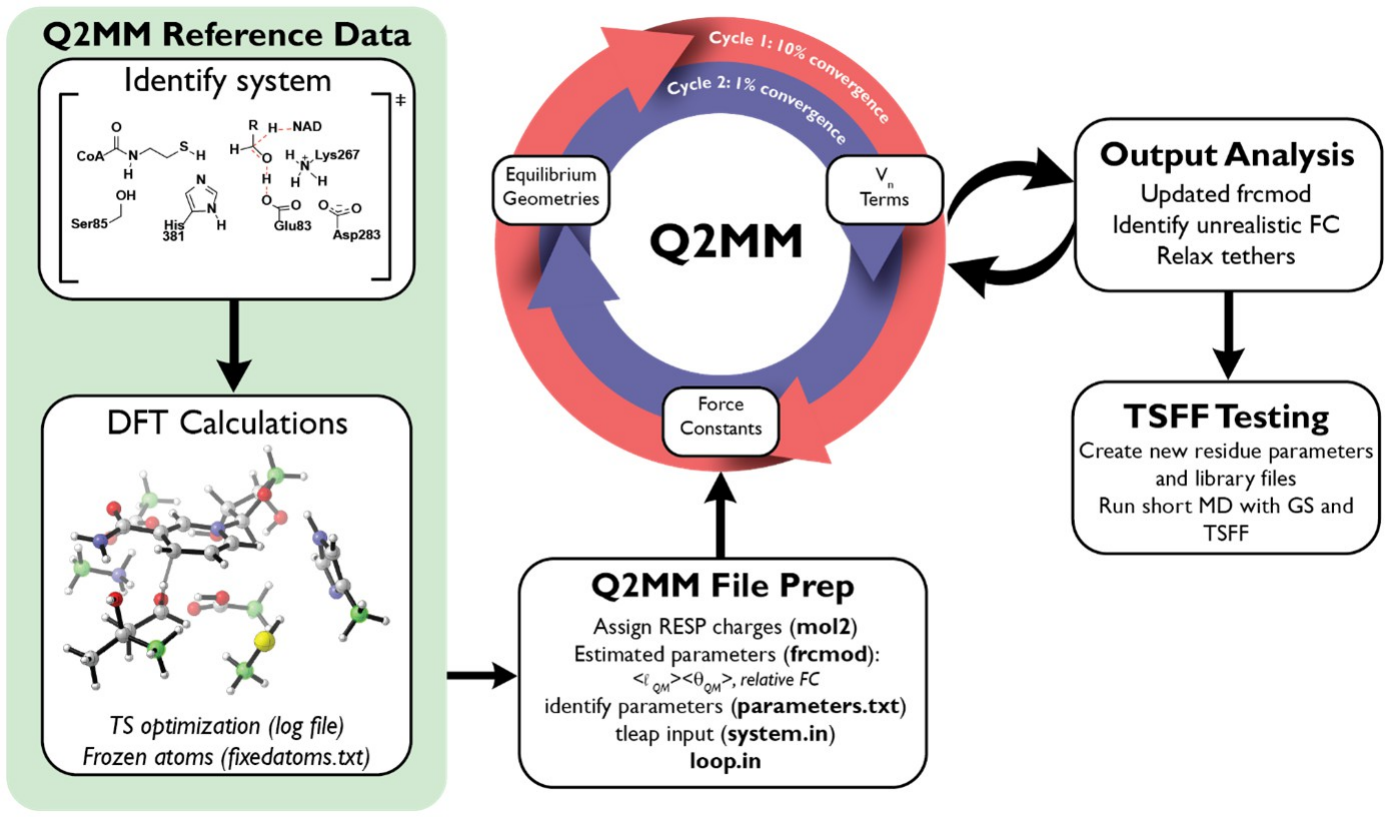
Q2MM workflow the parametrization of TSFFs for enzymatic reactions. The Q2MM system set up starts from the top left by first describing relevant system for the enzymatic reaction of interest, followed by reference DFT data calculation, which is included in the input files required by the program allowing it to iteratively develop a TSFF.

In order to develop a TSFF for an enzyme, the first step is to define a model system that includes the reactive species and the relevant parts of the protein involved in catalysis to generate the training data for the TS of this model system. Unlike in the case of the parameterization of small molecule TSFFs, where multiple simplified model systems are used [26], the full systems are used for the active site residues, substrates and cofactors selected in the case of the generation of a biomolecular TSFF. As a result, the atoms to be reparametrized need to be carefully selected to include the interactions relevant for the reaction under study. For the example discussed here, the QM/MM or theozyme [57] model incorporated the residues in the QM region derived from our previous studies [53, 54] of the mechanism of HMGR and shown in Fig 2, though other model systems were also explored [53]. Since this model system is derived from electronic structure calculations, only the most essential atoms should be included for efficiency of the fitting procedure even though the methodology is equally applicable to larger numbers of refitted atoms. A fixedatoms.txt (see S7 File in Supporting Information) file is created to include any atoms frozen in the electronic structure calculation (Fig 2, green atoms). Because the frozen atoms create unphysical Hessian elements, the weight of the Hessian values associated with these atoms are set to zero during the parameterization. Results of transition state optimizations, in a.log file, contain the energetic and geometric data that are used by Q2MM in the parametrization and are thereby included in the Q2MM input as reference. Currently, Q2MM supports interfaces to Gaussian [56] and Jaguar [49].log files as training data for the parameterization process.

**Fig 3:**
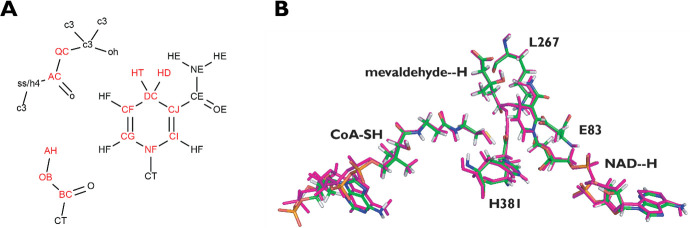
**(A)** Reassigned atom types for the second hydride transfer of *Pm*HMGR. **(B)** An overlay of geometries. Both systems show substrate, cofactor, and reparametrized protein atoms from electronic structure calculations (purple) and from the TSFF (atom colored). The RMSD for the 237 atoms in each system is 0.297 Å. Adapted from [64].

The.log file is also used to create a.mol2 file of the model system using the RESP protocol in Amber. The.mol2 file contains updated partial charges of all the atoms in the model system at the TS and is used throughout the parametrization. At this point, new atom types should be assigned to the atoms directly involved in the reaction, as their properties will be sufficiently different from that of the parent force field. The choice of the atoms to be reparametrized is system dependent and new atom types need to be assigned. For the case study presented here, the atoms that were reparametrized are shown in red in Fig 3A. This part of the procedure has similarities to the ideas of transfer learning in that parameters trained to a much larger dataset (standard parameters of the Amber force field) [21] and extensively validated in the literature are used as a starting point for retraining a much smaller subset for which smaller training data sets are available. It is a key difference from the development of TSFFs for transition metal catalyzed reactions [23–26] where there are usually no parameters available for the transition metal environment. As a result, a much larger training set is needed for the parameterization of transition states in transition metal catalyzed reactions to achieve a reliable TSFF [24, 42, 58]. Even though the number of atoms to be retrained is usually larger for the case of enzyme catalyzed reactions, the use of ideas from transfer learning makes the fitting procedure much more effective because the vast majority of atoms only undergoes minor perturbations in proceeding from the ground state to the transition state of the reaction.

The.mol2 file should also be used to generate the force field modification (frcmod) file, using Parmchk2 in the antechamber [59] suite of programs in Amber [52]. The.frcmod file needs to be updated accordingly to be used in Q2MM, examples of which can be found in the documentation on github. All parameters such as bonds, angles, and dihedrals for atoms directly involved in the reaction should be included in the.frcmod file. Initial values for the transition state parameters for the bond lengths and angles should be derived for the system at the TS to be consistent with the QM reference data. The proper estimation of the parameters minimizes the possibility of optimization to local nonphysical minima of the objective function and decreases the number of iterations required for parameterization. Force constants are initially set to standard values based on the General Amber Force Field (GAFF) [60]. To prevent over-parametrization, the force constants describing the dihedral angles are set to zero in the first part of the optimization when determining the bond distance and angle parameters. They are then set to values adapted from the GAFF force field and then optimized in a separate step as shown in Fig 2. The parameter.py module of Q2MM generates a list of a specified parameter type to be optimized that references the.frcmod file line and includes the range of values acceptable for that parameter type.

The input file, loop.in (S2 File in Supporting Information), for Q2MM files should contain the relevant information for an optimization cycle. The FFLD being read every cycle should be the given Amber.frcmod file (See S4 File in Supporting Information and the RDAT being read should be the Gaussian or Jaguar.log file. For CDAT, a tleap input file that calls the mol2 file and frcmod file of the model system and relevant Amber force fields should be created to generate a.prmtop and.inpcrd file that is used during the parameterization process. The optimization criteria of the penalty function are set in the loop.in file under the LOOP flag. Initially the penalty function can be set to a 10% convergence criterion, i.e. the penalty function does not change by more than 10% in successive iterations of the force field fitting procedure. The loop.in file can be submitted by >python loop.py loop.in.

After fitting of the partial charges of the transition state using the RESP protocol (Fig 2), they should remain unchanged throughout the course of the parameterization. Similarly, the Lennard-Jones parameters, which are fit to experimental values in the Amber force field, are not reparametrized [26]. The order of parameterization (Fig 2) is largely the same as in the case of TSFF parameterization for small molecules [26]. The force constants should be optimized first while ensuring that the optimized value stays above 32.2 kcal mol^-1^ Å^-2^ for bond distances and angles and 3.2 kcal mol^-1^ Å^-2^ for dihedrals to avoid undue distortions to optimized geometry. An interesting extension would be to implement the method of Seminario to more rapidly arrive at force constants fit to the Hessian only [61, 62], before the global reparametrization to achieve a fit to all input data. However, this extension has not yet been implemented. Subsequently, the bond length parameter can be refined to reflect the reference data. Bond angles can be optimized after the bond lengths while ensuring that the optimized values are within reasonable ranges. If the optimized angles deviate towards unphysical values for the atoms reparameterized, then these angle parameters value should be “tethered” to the reference data to prevent major deviations during optimization. Tethering is accomplished by controlling the changes of the parameter being optimized through increasing the weight value associated with that parameter. A higher tether weight should be used in the first round of optimization, then slowly decreased to zero in subsequent optimizations cycles. Finally, the V_n_ terms for the torsional potentials are fit to the Hessian data first before being further refined to further optimize the full penalty function. A second round of optimizations should be performed with a 1% convergence criterion for the penalty function to allow parameter refinement to be closer to the reference data parameters. Additional optimization cycles can be performed as needed until a suitable TSFF has been obtained that reproduces the training and test set of reference data to a sufficient level of accuracy as determined through plots of the reference values vs. values from the TSFF (see S6 File in the Supporting Information for an example). For enzymatic systems, a working TSFF is obtained when an optimization step changes the objective function by less than a 1% and the values and parameters are deemed suitable based on comparison with the results of the underlying electronic structure calculations and the standard force field parameters (for example plots, see S6 File in the Supporting Information).

The resulting force field should then be tested in a large-scale molecular dynamics simulations in conjunction with the ground state force field to describe the remainder of the protein (shown in gray in Fig 1). The TSFF will have to be parsed to generate new residue types that contain reparameterized atoms and new library files will need to be created to read into the leap module of Amber20 [52]. This could also involve setting conditions that allow the reacting atoms to have more than the standard amount of bonds in a system. Other important considerations are adjusting the timestep of the simulation to account for the vibrations of the reacting atoms and masking the SHAKE algorithm for hydrogens in the TSFF. A short MD simulation should then be performed to ensure that the total energy of the system remains stable with the TSFF in combination with the ground state FF that would be used for the rest of the enzyme.

## Application to *Pm*HMGR

This method described above was employed for the transition state of the second hydride transfer in *Pm*HMGR to demonstrate the application of Q2MM to generate an Amber-type TSFF for an enzyme catalyzed reaction. The reference data for the training of the TSFF were obtained from QM/MM calculations where the atoms indicated in Fig 2 were treated at the ONIOM-(B3LYP/6-31G(d,p):Amber) level of theory [53, 54]. This includes the side chains of H381, K267, D283 and E83 as well as the substrates and cofactor as shown in Fig 3 and the hmgrqm.log example file in the Supporting Information (S1 File). As the functional form of the underlying force file to which to fit the TSFF to, Amber99SB and GAFF for atoms on residues and substrates were used, respectively, as seen in the ts2.frcmod file. During parameterization, all atoms of the substrates and cofactor, along with the backbone and sidechains of the residues mentioned above, were included while calculating the MM data. Fig 3B shows and overlay of the geometries obtained from the electronic structure calculations and the TSFF, demonstrating the excellent agreement obtained by the much faster fore field calculations. As discussed earlier, the bonding character and partial charges of the atoms directly involved in the TS change in going from the ground to the transition state. Furthermore, the nicotinamide ring of the cofactor is dearomatized. To describe these perturbations, new atom types were introduced as indicated in Fig 3A. It is worth reemphasizing that the initial parameters for these new atom types were derived from the standard ground state Amber99SB parameters [21, 63] and then trained to reproduce the electronic structure results in the training data. In this specific case, only parameters directly associated with these atoms shown in red in Fig 3A (within 3 bonds) were reparameterized for the TSFF.

As shown in Fig 3B, the TSFF successfully reproduced the geometries around the reacting center of the active site and could successfully be incorporated into the rest of the enzyme that is treated with a traditional ground state force field. Using this, the enzyme could be simulated at the transition state on the microsecond timescale [64].

## Conclusions

In this contribution, we presented an automated workflow that combines the Q2MM method with transfer learning-type approaches for the generation of fast and accurate TSFFs for large biomolecular systems. Application of the workflow to the second hydride transfer of HMGR, an enzyme of high biomedical importance, shows that the transition state of this reaction can be accurately reproduced by the TSFF derived by this workflow.

The work presented here uses the philosophy of transfer learning in ML and applies it to the parametrization of TSFF by retraining of well validated existing force fields as opposed to creating completely new atom types and parameters, as has been done previously in the generation of small molecule TSFF that cover transition metal catalyzed reactions. Although the retraining of existing parameters rather than the definition of completely new elements simplifies the reparameterization, the number of atoms included is usually larger than in the case of small molecule catalysts. As a result of the high-dimensional optimization, special care needs to be taken to ensure that the Q2MM procedure does not lead to unphysical values for the TSFF. The results presented here are an early example for using exclusively electronic structure reference data and a much larger number of parameters adjusted in the biomolecular TSFF than in the earlier cases of small molecule TSFFs [25, 26] and the derivation of docking potentials [39, 40] using Q2MM. They show that the Q2MM approach can be used to parameterize a TSFF to simulate enzymes at the transition state ~10^4^ times faster than the underlying electronic structure methods, allowing for molecular dynamics simulation for system sizes and timescales well beyond the accessibility of DFT-based methods [64].

## Supporting information

S1 File DFT reference data for HMGR transition state (LOG). This Gaussian log file provides the reference QM data for the transition state needed to fit the TSFF.
(LOG)

S2 File Q2MM Input file (IN). This shows the Q2MM input file format.
(IN) Click here for additional data file.

S3 File HMGR transition state geometry (PDB). This is file shows the transition state geometry with the introduced atom names to distinguish theparameters of the ground state version from the transition state version of the atom of interest.
(PDB)

S4 File Transition state force field (FRCMOD). This is the final transition state force field file for Amber program. The parameters listed include new parameters for unique atom types and original parameters for atoms in the TSFF geometry that were not directly involved with the reaction.
(FRCMOD)

S5 File Partial charges for transition state (LIB). This file links the atom names (PDB) to the atom types (FRCMOD) while also providing the partial charges of each atom. The LIB file also provides a connectivity table for the atoms in the transition state which preserves the needed TS geometry.
(LIB)

S6 File Comparison of bond lengths, angles, dihedral angles and Hessian matrix elements from electronic structure calculations and TSFF. (PDF)

S7 File List of froz. (TXT)

## References

[1] Warshel A. Multiscale modeling of biological functions: from enzymes to molecular machines (Nobel Lecture). Angew Chem Intl Ed Engl 2014;53:10020–31. 10.1002/anie.201403689 PMC4948593 25060243

[2] Warshel A, Levitt M. Theoretical studies of enzymic reactions: Dielectric, electrostatic and steric stabilization of the carbonium ion in the reaction of lysozyme. J Mol Biol. 1976;103:227–49. doi: 10.1016/0022-2836(76)90311-9 985660

[3] Dauber-Osguthorpe P, Hagler AT. Biomolecular force fields: where have we been, where are we now, where do we need to go and how do we get there? J Comp Aid Des. 2019;33:133–203. 10.1007/s10822-018-0111-4 30506158

[4] Hagler AT. Force field development phase II: Relaxation of physics-based criteria… or inclusion of more rigorous physics into the representation of molecular energetics. J Comp Aid Des 2019;33(2):205–64. doi: 10.1007/s10822-018-0134-x 30506159

[5] Robustelli P, Piana S, Shaw DE. Developing a molecular dynamics force field for both folded and disordered protein states. Proc Natl Acad USA. 2018;115:E4758–E66. doi: 10.1073/pnas.1800690115 29735687 PMC6003505

[6] Vanommeslaeghe K, Guvench O. Molecular mechanics. Curr Pharm Des 2014;20:3281–92. doi: 10.2174/13816128113199990600 23947650 PMC4026342

[7] Lindorff-Larsen K, Maragakis P, Piana S, Eastwood MP, Dror RO, Shaw DE. Systematic validation of protein force fields against experimental data. PloS one. 2012;7:e32131. doi: 10.1371/journal.pone.0032131 22384157 PMC3285199

[8] Behler J. Perspective: Machine learning potentials for atomistic simulations. J Chem Phys. 2016;145:170901. doi: 10.1063/1.4966192 27825224

[9] Choudhary K, DeCost B, Tavazza F. Machine learning with force-field-inspired descriptors for materials: Fast screening and mapping energy landscape. Phys Rev Mat. 2018;2:083801. doi: 10.1103/physrevmaterials.2.083801 32166213 PMC7067064

[10] Ragoza M, Hochuli J, Idrobo E, Sunseri J, Koes DR. Protein-Ligand Scoring with Convolutional Neural Networks. J Chem Inf Mod. 2017;57:942–57. doi: 10.1021/acs.jcim.6b00740 28368587 PMC5479431

[11] Jordan MI, Mitchell TM. Machine learning: Trends, perspectives, and prospects. Science. 2015;349:255–60. doi: 10.1126/science.aaa8415 26185243

[12] Behler J. First Principles Neural Network Potentials for Reactive Simulations of Large Molecular and Condensed Systems. Angew Chem Intl Ed. 2017;56:12828–40. doi: 10.1002/anie.201703114 28520235

[13] Behler J, Parrinello M. Generalized neural-network representation of high-dimensional potential-energy surfaces. Phys Rev Lett. 2007;98:146401. doi: 10.1103/PhysRevLett.98.146401 17501293

[14] Yao K, Herr JE, Toth DW, McKintyre R, Parkhill J. The TensorMol-0.1 model chemistry: A neural network augmented with long-range physics. Chem Sci. 2018;9:2261–9. doi: 10.1039/c7sc04934j 29719699 PMC5897848

[15] Schran C, Uhl F, Behler J, Marx D. High-dimensional neural network potentials for solvation: The case of protonated water clusters in helium. J Chem Phys. 2018;148:102310. doi: 10.1063/1.4996819 29544280

[16] Senn HM, Thiel W. QM/MM Methods for Biomolecular Systems Angew Chem Intl Ed 2009;48:1198–229. doi: 10.1002/anie.200802019 19173328

[17] König P, Hoffmann M, Frauenheim T, Cui Q. A critical evaluation of different QM/MM frontier treatments with SCC-DFTB as the QM method. J Phys, Chem B. 2005;109:9082–95. doi: 10.1021/jp0442347 16852081

[18] Chung LW, Sameera W, Ramozzi R, Page AJ, Hatanaka M, Petrova GP, et al. The ONIOM method and its applications. Chem Rev. 2015;115:5678–796. doi: 10.1021/cr5004419 25853797

[19] Eksterowicz JE, Houk KN. Transition-state modeling with empirical force fields. Chem Rev 1993;93:2439–61.

[20] Garbisch EW Jr, Schildcrout SM, Patterson DB, Sprecher CM. Strain Effects. II. Diimide Reductions of Olefins. J Am Chem Soc. 1965;87:2932–44.

[21] Ponder JW, Case DA. Force fields for protein simulations. Adv Prot Chem. 2003;66:27–85. doi: 10.1016/s0065-3233(03)66002-x 14631816

[22] Norrby P-O, Åkermark B, Haeffner F, Hansson S, Blomberg M. Molecular Mechanics (MM2) Parameters for the (eta-3-allyl) palladium Moiety. J Am Chem Soc. 1993;115:4859–67.

[23] Rosales A, Ross SP, Helquist P, Norrby P-O, Sigman MS, Wiest O. Transition State Force Field for the Asymmetric Redox Relay Heck Reaction. J Am Chem Soc 2020;142:9700–7. doi: 10.1021/jacs.0c01979 32249569 PMC7304536

[24] Rosales AR, Wahlers J, Limé E, Meadows RE, Leslie KW, Savin R, et al. Rapid virtual screening of enantioselective catalysts using CatVS. Nature Catalysis. 2019;2:41–5.

[25] Hansen E, Rosales AR, Tutkowski B, Norrby P-O, Wiest O. Prediction of Stereochemistry using Q2MM. Acc Chem Res. 2016;49:996–1005. doi: 10.1021/acs.accounts.6b00037 27064579 PMC4879660

[26] Rosales AR, Quinn TR, Wahlers J, Tomberg A, Zhang X, Helquist P, et al. Application of Q2MM to predictions in stereoselective synthesis. Chem Comm. 2018;54:8294–311. doi: 10.1039/c8cc03695k 29971313 PMC6069518

[27] Warshel A. Computer modelling of chemical reactions in enzymes and solutions. New York: Wiley-Interscience 1991.

[28] Åqvist J, Warshel A. Simulation of enzyme reactions using valence bond force fields and other hybrid quantum/classical approaches. Chem Rev. 1993;93:2523–44.

[29] Florian J. Comment on Molecular Mechanics for Chemical Reactions. J Phys Chem A. 2002;106:XX.

[30] Truhlar DG. Reply to Comment on Molecular Mechanics for Chemical Reactions. J Phys Chem A. 2002;106:5048–50.

[31] Li Y, Li H, Pickard FC, Narayanan B, Sen FG, Chan MKY, et al. Machine Learning Force Field Parameters from Ab Initio Data. J Chem Theor Comp. 2017;13:4492–503. doi: 10.1021/acs.jctc.7b00521 28800233 PMC5931379

[32] Van Duin ACT, Dasgupta S, Lorant F, Goddard WA. ReaxFF: A reactive force field for hydrocarbons. J Phys Chem A. 2001;105:9396–409.

[33] Lim VT, Mobley D. Benchmark Assessment of Molecular Geometries and Energies from Small Molecule Force Fields. ChemRxiv. 2020; doi: 10.12688/f1000research.27141.1 33604023 PMC7863993

[34] Wang LP, McKiernan KA, Gomes J, Beauchamp KA, Head-Gordon T, Rice JE, et al. Building a More Predictive Protein Force Field: A Systematic and Reproducible Route to AMBER-FB15. J Phys Chem B. 2017;121:4023–39. doi: 10.1021/acs.jpcb.7b02320 28306259 PMC9724927

[35] Wang LP, Martinez TJ, Pande VS. Building force fields: An automatic, systematic, and reproducible approach. J Phys Chem Lett. 2014;5:1885–91. doi: 10.1021/jz500737m 26273869 PMC9649520

[36] Shi Y, Xia Z, Zhang J, Best R, Wu C, Ponder JW, et al. Polarizable atomic multipole-based AMOEBA force field for proteins. J Chem Theor Comp. 2013;9:4046–63. doi: 10.1021/ct4003702 24163642 PMC3806652

[37] Donoghue PJ, Helquist P, Norrby P-O, Wiest O. Prediction of enantioselectivity in rhodium catalyzed hydrogenations. J Am Chem Soc. 2009;131:410–1. doi: 10.1021/ja806246h 19140780

[38] Limé E, Lundholm MD, Forbes A, Wiest O, Helquist P, Norrby P-O. Stereoselectivity in asymmetric catalysis: The case of ruthenium-catalyzed ketone hydrogenation. J Chem Theor Comp. 2014;10:2427–35. doi: 10.1021/ct500178w 26580763

[39] Rydberg P, Hansen SM, Kongsted J, Norrby P-O, Olsen L, Ryde U. Transition-state docking of flunitrazepam and progesterone in cytochrome P450. J Chem Theor Comp. 2008;4:673–81. doi: 10.1021/ct700313j 26620942

[40] Rydberg P, Olsen L, Norrby P-O, Ryde U. General transition-state force field for cytochrome P450 hydroxylation. J Chem Theor Comp. 2007;3:1765–73. doi: 10.1021/ct700110f 26627620

[41] Limé E, Norrby P-O. Improving the Q2MM method for transition state force field modeling. J Comp Chem. 2015;36:244–50. doi: 10.1002/jcc.23797 25430788

[42] Donoghue PJ, Helquist P, Norrby P-O, Wiest O. Development of a Q2MM force field for the asymmetric rhodium catalyzed hydrogenation of enamides. J Chem Theor Comp 2008;4:1313–23. doi: 10.1021/ct800132a 26631706

[43] Norrby P-O, Liljefors T. Automated molecular mechanics parameterization with simultaneous utilization of experimental and quantum mechanical data. J Comp Chem. 1998;19:1146–66.

[44] Press WH, Teukolsky SA, Vetterling WT, Flannery BP. Downhill Simplex Method in Multidimensions. New York: Cambridge University Press; 1992 1992.

[45] Huang L, Roux B. Automated force field parameterization for nonpolarizable and polarizable atomic models based on ab initio target data. J Chem Theor Comp. 2013;9:3543–56. 10.1021/ct4003477 PMC3819940 24223528

[46] Wu JC, Chattree G, Ren P. Automation of AMOEBA polarizable force field parameterization for small molecules. Theor Chem Acc. 2012;131:1138. doi: 10.1007/s00214-012-1138-6 22505837 PMC3322661

[47] Maple JR, Hwang MJ, Stockfisch TP, Dinur U, Waldman M, Ewig CS, et al. Derivation of class II force fields. I. Methodology and quantum force field for the alkyl functional group and alkane molecules. J Comp Chem. 1994;15:162–82.

[48] Halgren TA. Merck molecular force field. I. Basis, form, scope, parameterization, and performance of MMFF94. J Comp Chem. 1996;17:490–519.

[49] Schrödinger Release 2020–1: Schrödinger, LLC, New York, NY, 2020.

[50] Hagler AT, Ewig CS. On the use of quantum energy surfaces in the derivation of molecular force fields. Comp Phys Comp. 1994;84:131–55.

[51] Madarász A, Berta D, Paton RS. Development of a true transition state force field from quantum mechanical calculations. J Chem Theor Comp. 2016;12:1833–44. doi: 10.1021/acs.jctc.5b01237 26925858

[52] Case DA, Belfon K, Ben-Shalom IY, Brozell SR, Cerutti DS, Cheatham I, T.E.;, et al. AMBER 20. University of California, San Francisco, CA, USA2020.

[53] Haines BE. Computational Studies on the Mechanism of HMG-CoA Reductase and the Grignard S_RN_1 reaction. PhD Thesis [PhD Thesis]: Notre Dame 2014.

[54] Haines BE, Steussy C. N., Stauffacher C. V., Wiest O. Molecular Modeling of the Reaction Pathway and Hydride Transfer Reactions of HMG-CoA Reductase. Biochemistry. 2012;51:7983–95. doi: 10.1021/bi3008593 22971202 PMC3522576

[55] Haines BE, Wiest O., Stauffacher C. V. The Increasingly Complex Mechanism of HMG CoA Reductase. Acc Chem Res. 2013;46:2416–26. doi: 10.1021/ar3003267 23898905 PMC4118817

[56] Frisch MJT, G. W.; Schlegel, H. B.; Scuseria, G. E.; Robb, M. A.; Cheeseman, J. R.; Scalmani, G.; et al. Gaussian 16 Revision C.01. Wallingford, CT2016.

[57] Tantillo D, Houk KN. Theozymes and compuzymes: theoretical models for biological catalysis. Curr Opin Chem Biol 1998;2:743–50. doi: 10.1016/s1367-5931(98)80112-9 9914196

[58] Wahlers J, Maloney M, Salahi F, Rosales AR, Helquist P, Norrby P-O, et al. Stereoselectivity Predictions for the Pd-Catalyzed 1, 4-Conjugate Addition Using Quantum-Guided Molecular Mechanics. J OrgChem. 2021;86:5660–7. doi: 10.1021/acs.joc.1c00136 33769065 PMC8103656

[59] Wang J, Wang W, Kollman PA, Case DA. Automatic atom type and bond type perception in molecular mechanical calculations. J Mol Graph Mod 2006;25:247–60. doi: 10.1016/j.jmgm.2005.12.005 16458552

[60] Wang J, Wolf RM, Caldwell JW, Kollman PA, Case DA. Development and testing of a general amber force field. J Comp Chem. 2004;25:1157–74. doi: 10.1002/jcc.20035 15116359

[61] Allen AE, Payne MC, Cole DJ. Harmonic force constants for molecular mechanics force fields via Hessian matrix projection. J Chem Theor Comp. 2018;14:274–81. doi: 10.1021/acs.jctc.7b00785 29161029

[62] Seminario JM. Calculation of intramolecular force fields from second-derivative tensors. Int J Quant Chem 1996;60:1271–7.

[63] Pavelites JJ, Gao J, Bash PA, Mackerell AD Jr. A molecular mechanics force field for NAD+ NADH, and the pyrophosphate groups of nucleotides. J Comp Chem. 1997;18:221–39.

[64] Quinn TR, Steussy CN, Haines BE, Lei J, Wang W, Sheong FK, et al. Microsecond timescale MD simulations at the transition state of Pm HMGR predict remote allosteric residues. Chem Sci 2021;12:6413–8. doi: 10.1039/d1sc00102g 34084441 PMC8115266

