# Supplementary material for: Automated fitting of transition state force fields for biomolecular simulations
Source: PLoS One. 2022 Mar 10;17(3):e0264960. doi: 10.1371/journal.pone.0264960 (PMC8912266; doi:10.1371/journal.pone.0264960)
Supplement: S6 File — (PDF) [file pone.0264960.s006.pdf]

Comparison of bond distances (A), bond angles (B), dihedral angles (C) and Hessian matrix elements (D) from electronic structure calculations and fitted TSFF

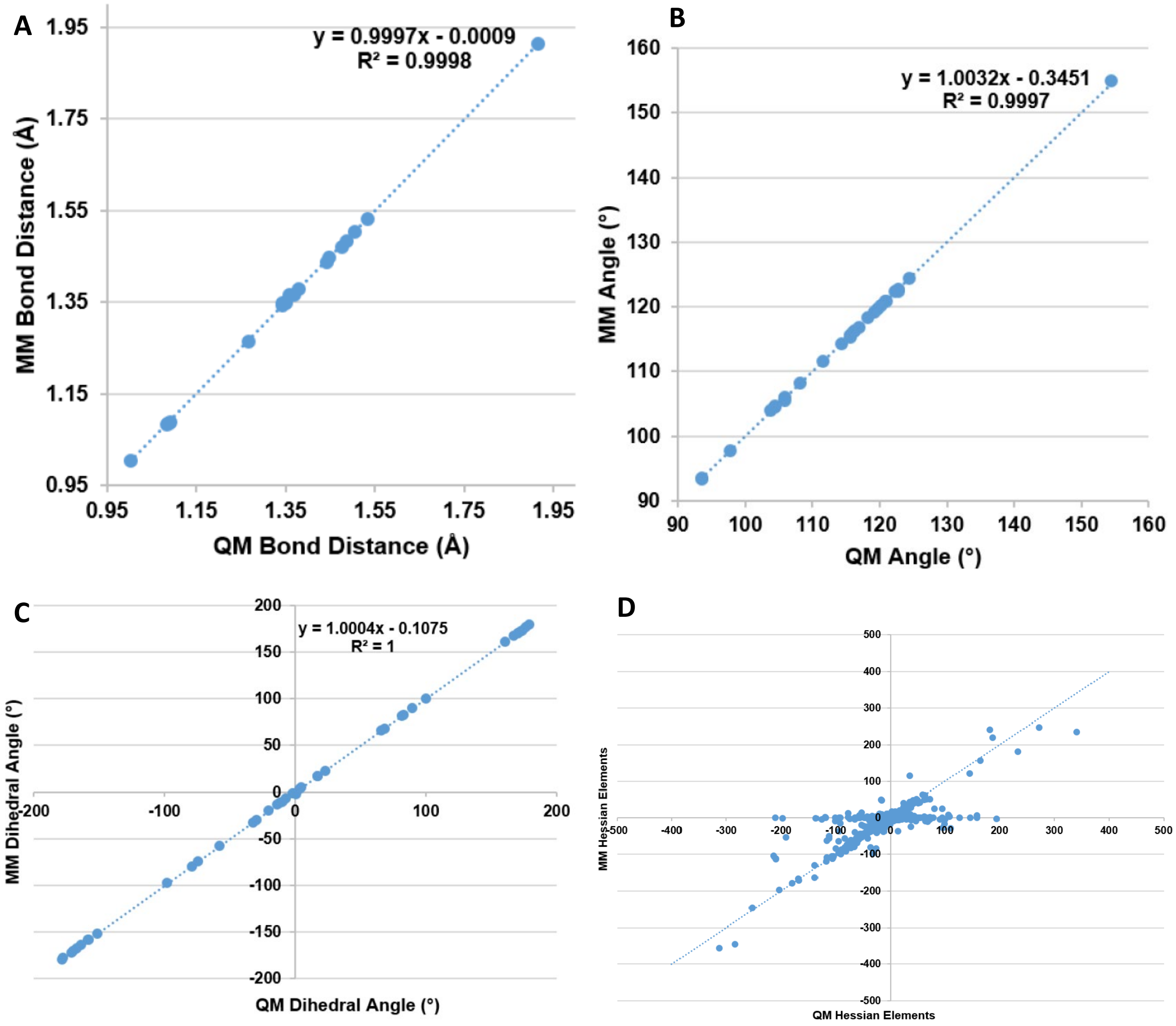

Note that there are a significant number of hessian matrix elements that a set to 0 in the optimized TSFF. For details, see [1, 2]

1. Norrby P-O, Liljefors T. Automated molecular mechanics parameterization with simultaneous utilization of experimental and quantum mechanical data. J Comp Chem. 1998;19:1146-66.

2. Limé E, Norrby P-O. Improving the Q2MM method for transition state force field modeling. J Comp Chem. 2015;36:244-50.
